# MRI assessment of altered dynamic changes in liver haemodynamics following a meal challenge in compensated cirrhosis

**DOI:** 10.1186/s41747-018-0056-3

**Published:** 2018-09-26

**Authors:** Eleanor F. Cox, Naaventhan Palaniyappan, Guruprasad P. Aithal, Indra N. Guha, Susan T. Francis

**Affiliations:** 1 Sir Peter Mansfield Imaging Centre, School of Physics & Astronomy, University of Nottingham, Nottingham, UK; 2 NIHR Nottingham BRC, Nottingham University Hospitals NHS Trust and the University of Nottingham, Nottingham, UK; 3 Nottingham Digestive Diseases Centre, School of Medicine, University of Nottingham, Nottingham, UK

**Keywords:** Arterial spin labelling (ASL), Liver cirrhosis, Magnetic resonance imaging (MRI), Perfusion, T2* mapping

## Abstract

**Background:**

The aim was to use magnetic resonance imaging (MRI) to dynamically assess postprandial changes in hepatic and collateral blood flow, liver perfusion and oxygenation in healthy participants and patients with liver disease with compensated cirrhosis (CC).

**Methods:**

We evaluated blood flow in the portal vein, hepatic artery and azygos vein (using phase-contrast MRI), liver perfusion (using arterial spin labelling) and blood oxygenation (using transverse relaxation time [T2*] mapping). Measures were collected at baseline and at 6–7-min intervals from 20 to 65 min following a test meal (440 mL, 660 kcal) in 10 healthy participants and 10 patients with CC.

**Results:**

In healthy participants, we observed a significant postprandial increase in portal vein flow from baseline (+ 137 ± 26% (mean ± standard deviation), *p* < 0.001) coupled with a reduction in hepatic artery flow from baseline (− 30 ± 18%, *p* = 0.008), reflecting the hepatic artery buffer response. In patients with CC, a lower but still significant increase in portal vein flow (67 ± 50%, *p* = 0.014) was observed, without a clear hepatic artery buffer response. Healthy participants showed a significant increase in postprandial liver perfusion (138 ± 75%, *p* < 0.001), not observed in patients with CC. There was no change in liver T2* for either group.

**Conclusions:**

Postprandial changes in liver perfusion, oxygenation and hepatic and collateral circulation can be measured noninvasively using MRI. Differences between healthy participants and patients with CC were shown, which may help stratify liver cirrhosis in patients.

## Key points

- Phase-contrast flow measurements in healthy participants reflect the hepatic artery buffer response
- The hepatic artery buffer response was reduced in patients with compensated cirrhosis
- A postprandial increase in liver tissue perfusion was observed in healthy participants, not in patients

## Background

Portal hypertension is the most critical complication of liver cirrhosis, in which alterations in hepatic architecture lead to increased portal blood flow and intrahepatic resistance. Food intake increases splanchnic blood flow (postprandial hyperaemia) and the postprandial response varies with disease. In cirrhosis, the inability of the hepatic microcirculation to vasodilate in response to postprandial hyperaemia provokes a rise in collateral blood flow and portal pressure [1]. However, the mechanism remains unclear [2].

The noninvasive assessment of sequential postprandial measurements of collateral blood flow, portal haemodynamics and oxygenation could provide insights into the capacity for dynamic change within the liver. Previous studies [1–6] have assessed individual components of this dynamic change, but have not comprehensively assessed the dynamic change in flow, perfusion and oxygenation within the liver.

Potential methods to assess the postprandial response include portal pressure assessment using the hepatic venous pressure gradient [1–4]. However, this approach is invasive and not widely available, thus repeated dynamic postprandial measurements are not feasible in clinical practice. Doppler ultrasound studies measuring portal vein flow have shown that the postprandial response is blunted in patients with chronic liver disease compared with healthy participants [5, 6], but these measures have poor reproducibility, with a high intra-observer and inter-observer variability [6–9]. Phase contrast (PC) magnetic resonance imaging (MRI) data can be collected for two-dimensional flow in which individual vessels are interrogated [10–12] or, more recently, for four-dimensional flow, allowing the assessment of several vessels in a single scan [13], though this comes at the cost of a significantly lengthened acquisition time [14]. PC-MRI methods have been shown to provide improved reproducibility over Doppler ultrasound [10–12, 15]. Previous PC-MRI studies of the postprandial response have shown either no postprandial difference between healthy participants and patients with liver disease [10] or reduced postprandial portal venous flow in patients with portal hypertension compared to healthy participants [16]. Liver stiffness measurements (LSM) using transient elastography or magnetic resonance elastography have shown a postprandial increase in liver stiffness in healthy participants, which is more pronounced and progressively increased with liver disease severity [17, 18]. However, since LSM have contributions from both a static component reflecting hepatic fibrosis progression and a dynamic component reflecting extrinsic perfusion changes, interpreting LSM alone to assess underlying changes in postprandial portal flow and intrahepatic resistance is challenging.

Multiparametric liver MRI permits the repeat assessment of haemodynamic and oxygenation measures within a single scan session. Hepatic and collateral blood flow, liver perfusion and liver oxygenation can be measured using PC-MRI, arterial spin labelling (ASL) and transverse relaxation time (T2*) mapping through blood oxygen-level-dependent MRI, respectively. Independent changes in blood flow to the liver and oxygen consumption within the liver will influence liver blood oxygenation levels, which will be reflected by changes in the liver T2*, and measurement of blood flow and perfusion allows these contributions to be separated. Furthermore, since MRI is noninvasive, sequential postprandial assessments are feasible and acceptable.

To date, only a few studies have assessed haemodynamic components of the postprandial response of the liver using MRI in healthy participants [13, 16, 19–22] or in patients with liver cirrhosis [13, 16, 19, 20]. However, it is difficult to compare results between these studies because a wide variety of meals have been used and measurements have been made at different times following ingestion of the meal, ranging from 15 to 150 min.

Our aim was to evaluate the sequential dynamic postprandial changes in hepatic and collateral circulation, liver perfusion and oxygenation using quantitative MRI in healthy participants and patients with compensated cirrhosis (CC).

## Methods

### Study population

A total of 10 healthy subjects (5 male and 5 female; age 30 ± 10 years (mean ± standard deviation), range 22–56 years) and 10 patients with CC (8 male and 2 female; age 59 ± 11 years, range 38–68 years) were recruited. Healthy participants had no history of liver disease or significant alcohol consumption. Enrolled patients had histological or radiological evidence of liver cirrhosis. Exclusion criteria included previous liver decompensation (ascites, variceal bleeding or overt encephalopathy), hepatocellular carcinoma, orthotopic liver transplantation, portal or hepatic vein thrombosis, beta-blocker therapy, contraindications for MRI, age below 18 years, pregnancy and portal vein thrombosis.

The study was carried out according to the principles of the Declaration of Helsinki. Approval for the healthy participant study was granted by the local research ethics committee. Approval for the patient study was granted by East Midlands Research Ethics Committee – Nottingham 1 (Ref 12/EM/0401). Written informed consent was obtained from all participants.

### Study design

**Fig. 1:**
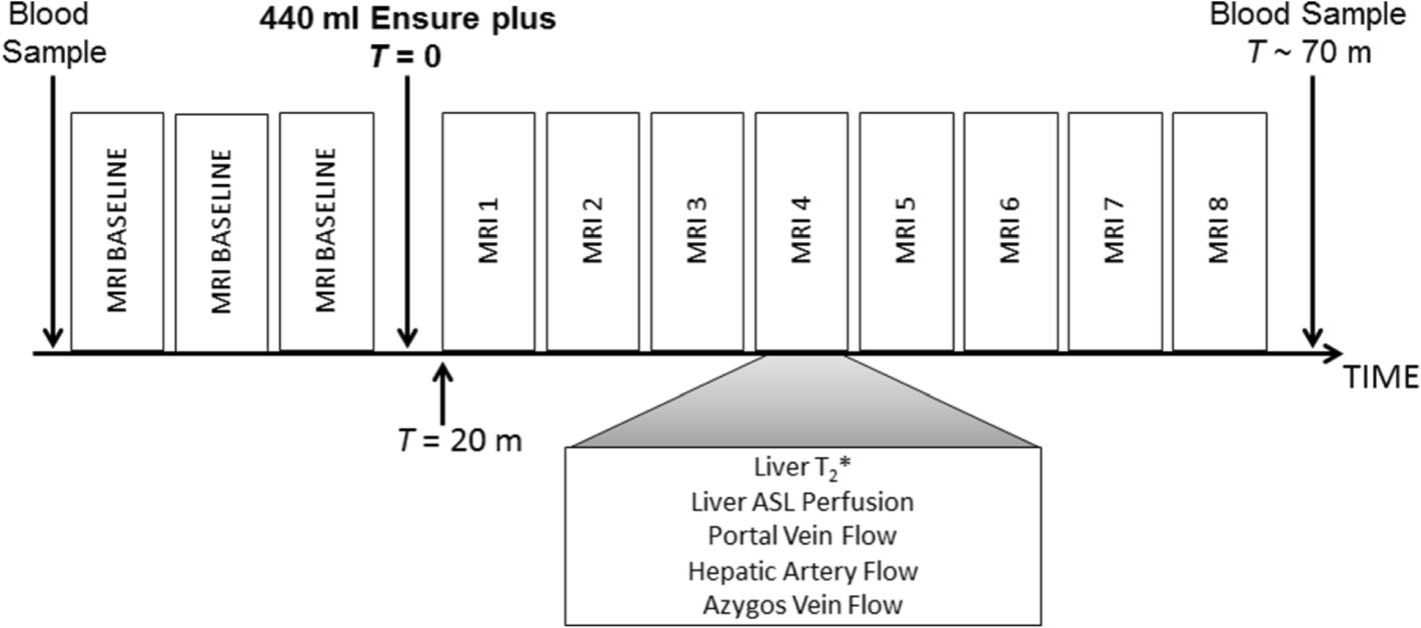
Magnetic resonance imaging (MRI) protocol comprising serial measurements. Measurements were collected in triplicate at baseline and then repeated from time (*T*) = 20–65 min following meal ingestion. Measurements were collected to assess blood flow to the liver and collateral flow (using phase contrast sequences), liver tissue perfusion (using the arterial spin labelling (ASL) technique) and liver transverse relaxation time (T2*), an indirect marker of oxygenation

The study design is illustrated in Fig. 1. All participants were scanned following a 6-h fast. MRI scans were carried out between 8 am and 12 pm, except for one healthy participant and one patient with CC who were scanned in the afternoon. Baseline MRI measurements of localiser scans followed by assessment of hepatic and collateral blood flow, liver perfusion and liver T2* in triplicate were initially acquired. Thereafter, participants were taken out of the scanner and they then drank a 440-mL liquid test meal composed of 22 g fat, 89 g carbohydrate, and 28 g protein for a total of 660 kcal (Ensure Plus, Abbott, Utrecht, The Netherlands). The onset of meal ingestion was taken as time *T* = 0 min and the meal was ingested within 5 min. Subjects then re-entered the scanner to allow planning localiser scans to again be collected prior to repeating the scans at intervals of about 6-7 min (breathing-rate dependent) from *T* = 20–65 min after meal ingestion. This period was chosen as previous studies [1–3, 5, 17, 23–30] showed peak postprandial response occurred within this time.

### Enhanced liver fibrosis score and liver stiffness measurement

Blood samples were obtained before and immediately after MRI scanning, collected outside of the scanner. Samples were analysed for alanine transaminase (ALT), alkaline phosphatase (ALP), gamma-glutamyl transferase (GGT) and bilirubin. In addition, levels of tissue inhibitor of matrix metalloproteinase 1 (TIMP1), hyaluronic acid (HA), and aminoterminal peptide of procollagen III (P3NP) were measured at an independent reference laboratory (iQur Limited, London, UK) in order to calculate an enhanced liver fibrosis (ELF) score [31].

Healthy participants also took part in a separate study session on a different day in which LSM was performed at baseline and at 15-min intervals following the ingestion of the test meal, up to 60 min. LSM was performed by experienced operators using FibroScan® (Echosens, Paris, France) with 10 readings obtained to calculate median LSM. Patients did not attend on a different day for the LSM because the postprandial change in LSM during a meal has been reported across varying degrees of liver fibrosis [17]. We attempted to measure the LSM in the patients before the meal challenge and after 60 min in the MRI scanner.

### MRI acquisition

MRI scans were acquired on a 3-T Achieva scanner (Philips Medical Systems, Best, The Netherlands) using a multi-transmit body coil (16-channel sensitive encoding torso receive coil). First, multi-slice balanced turbo field echo data were acquired in three orthogonal planes to locate the liver and vessels.

#### Hepatic and collateral blood flow

PC-MRI data were collected to assess hepatic (portal vein, hepatic artery) and collateral (azygos vein) blood flow. A turbo field echo sequence with imaging slice placed perpendicular to each vessel was used to collect *N* phases across the cardiac cycle (turbo factor 4–6, heart-rate dependent, Table 1). The portal vein was measured before its split into right and left portal veins; the hepatic artery was measured after splitting from the coeliac artery; and the azygos vein was measured between the azygos arch and the accessory hemiazygos vein. Each PC-MRI measurement was acquired during a 15–20-s breath hold.

**Table 1:** Magnetic resonance parameters for blood flow assessment using phase contrast magnetic resonance imaging, liver perfusion measures using arterial spin labelling (ASL) and transverse relaxation (T2*) mapping of the liver

Parameter	Phase contrast			ASL	T2*
	Portal vein	Hepatic artery	Azygos vein		
Reconstructed voxel (mm^3^)	1.17 × 1.17 × 6	1.17 × 1.17 × 6	1.17 × 1.17 × 6	3 × 3 × 5	1 × 1 × 8
Repetition time (ms)	8.4	5.6	7.5	2.9	365
Echo time (ms)	3.7	3.2	3.7	1.45	2.5
Flip angle (°)	25	25	25	45	30
SENSE factor	2	2	2	2	2.5
Velocity encoding (cm/s)	50	Baseline 100 Postprandial 150	50	NA	NA
Phases (number)	20	30	20	NA	NA

*SENSE* sensitivity encoding, *NA* not available

#### Liver perfusion

Multiphase flow-sensitive alternating inversion recovery ASL data (30 selective/non-selective pairs) were collected with a balanced fast field echo readout (centric half-Fourier acquisition, shot duration 130 ms, Table 1) in a sagittal slice through the right lobe of the liver, avoiding major vessels. Data were collected with a respiratory trigger delay of 500 ms prior to the ASL labelling. Labelling was followed by Look-Locker sampling (initial delay 100 ms, subsequent readout spacing 350 ms) with six readout phases collected to span post-label delay times of 100–1850 ms [32]. A base magnetization (M_0_) image was also collected for perfusion quantification.

#### Liver T2* mapping

A multi-echo fast field echo sequence was used to obtain 8 contiguous axial slices (echo spacing 2.5 ms, 12 echoes, Table 1). Each T2* measurement was collected during a 17-s breath hold.

### Magnetic resonance imaging analysis

#### Hepatic and collateral blood flow

For each vessel, the mean vessel cross-sectional area (mm^2^), velocity (cm/s) and hence, flow (mL/s), across the cardiac cycle was calculated using Q-flow software (Philips Medical Systems, Best, The Netherlands). Total hepatic blood flow (THBF) was calculated from the sum of the portal vein and hepatic artery blood flow. Azygos vein flow was used to indicate collateral blood flow.

#### Liver perfusion

In-house programs were used to quantify perfusion (mL/100 g/min) from ASL data (MATLAB, The MathWorks Inc., Natick, MA, USA). Individual difference images (selective minus non-selective) were calculated and averaged to form perfusion-weighted difference (ΔM) maps for each post-label delay. Non-selective images were used to perform a voxel-wise fit for longitudinal relaxation time, T1. A liver mask was formed from the base M_0_ image and applied to the T1 map to form a histogram of liver T1 values. Histogram analysis assessed the mode (μ) and standard deviation (SD) of the T_1_ distribution across the liver. A liver tissue mask was formed from voxels with T_1_ = μ ± SD, and a liver vessel mask from voxels with T1 > (μ + SD). Mean values of ΔM, T1 and M_0_ for liver tissue were used in an iterative model [33, 34] to calculate liver tissue perfusion and arrival time of the labelled blood to the liver tissue, assuming a blood T1 relaxation time of 1.55 s at 3 T.

#### Liver T2* mapping

The log of the exponential transverse signal decay was fit for T2* (MATLAB, The MathWorks Inc., Natick, MA, USA). A histogram of liver T2* values was formed and the mode and full width at half maximum (FWHM) of the T2* distribution computed.

### Statistics

Statistical analysis was performed using SPSS software version 21(IBM, New York, USA), with *p* values < 0.05 considered as statistically significant. Normality was tested using the Shapiro-Wilk test. Normal data are given as mean ± SD, else median and interquartile range is given. Groups were compared using the independent samples two-tailed *t* test if data were normally distributed, else the Mann-Whitney *U* test. Changes between baseline and postprandial time points were analysed using the paired *t* test when time-points were normally distributed, else the related-samples Wilcoxon signed rank test. The Bonferroni correction for multiple comparisons was applied. Within-session baseline repeatability of MRI measurements was calculated using the coefficient of variation (CoV) and intra-class correlation (ICC, two-way mixed, absolute agreement, average measures).

## Results

Clinical and biochemical parameters of the patients with CC are presented in Table 2. Diagnosis of cirrhosis was established either from a liver biopsy (*n* = 9) or an abdominal ultrasound (*n* = 1). All patients had a Childs-Pugh score < 6 points (Childs-Pugh A). Nine patients underwent oesophagogastroduodenoscopy within 2 years of the study. Five patients had no evidence of gastro-oesophageal varices and four patients had evidence of small grade-1 varices.

**Table 2:** Clinical and laboratory data for the patients with compensated cirrhosis

Variables	Patients with compensated cirrhosis (*n* = 10)
Aetiology (*n*)	
Alcohol	6
Non-alcoholic fatty liver disease	2
Chronic hepatitis C	1
Haemochromatosis	1
Gastro-oesophageal varices (*n*)	
No varices	5
Grade 1	4
Albumin (g/L)	40.5 ± 3.7
Prothrombin time (s)	10.6 ± 0.7
Platelet count (× 10^9^/L)	229.6 ± 122.9
Serum sodium (mmol/L)	139.0 ± 3.3
Serum creatinine (μmol/L)	72.4 ± 16.4

Data are numbers (frequencies) or mean ± standard deviation

*n* number

### Baseline measures

**Fig. 2:**
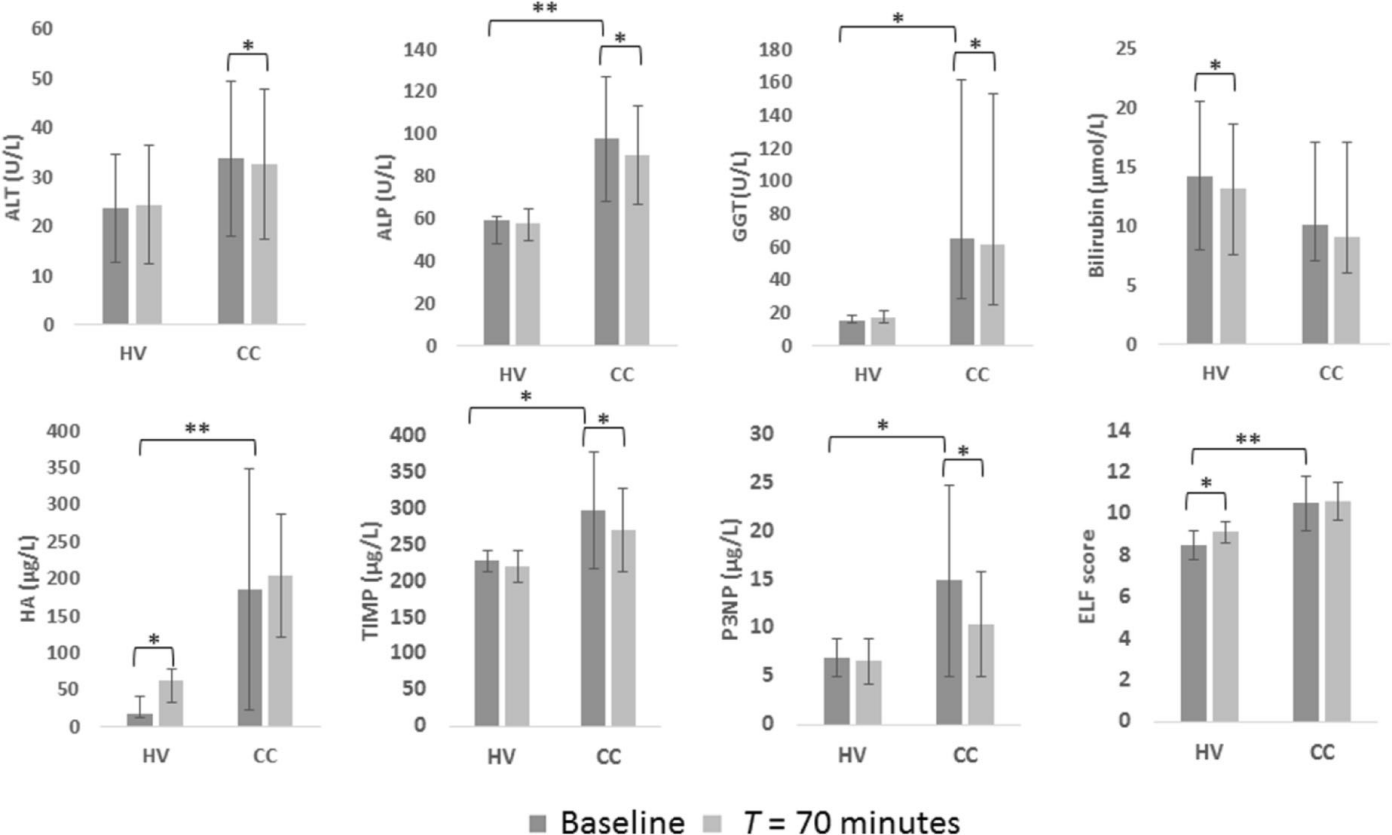
Serum markers at baseline and at time (*T*) = 70 min following meal ingestion in healthy participants (HV) and patients with compensated cirrhosis (CC). *ALT* alanine transaminase; *ALP* alkaline phosphatase; *GGT* gamma-glutamyl transferase; *HA* hyaluronic acid; *TIMP* tissue inhibitor of matrix metalloproteinase 1; *P3NP* aminoterminal peptide of procollagen III; *ELF* enhanced liver fibrosis. *p < 0.05, **p < 0.01

Figure 2 provides serum levels of ALT, ALP, GGT and bilirubin at baseline in healthy participants and patients with CC. All healthy participants had normal serum levels at baseline, while serum levels of ALP and GGT were significantly higher in Patients with CC. As expected, baseline ELF score and constituent serum levels were significantly higher in Patients with CC compared to healthy participants (Fig. 2). Baseline LSM for healthy participants was 4.1 kPa (range 3.6–4.4).

Baseline MRI measures are listed in Table 3 with the associated CoV and ICC. The CoV of vessel flow parameters were between 2.5 and 15.4%. The area of the portal vein was larger in patients with CC compared to healthy participants, whilst portal vein blood flow velocity was similar between groups, resulting in significantly higher portal vein blood flow in patients with CC than in healthy participants. There were no differences between groups in velocity, area or flow in the hepatic artery, thus the higher THBF in patients with CC was dominated by increased portal vein flow. Azygos vein flow was significantly higher in patients with CC than in healthy participants. There was no difference between groups in liver tissue perfusion, whilst arrival time of blood to the liver tissue was shorter in patients with CC compared with healthy participants (Table 3). There was no difference in liver tissue T2* between groups, despite an excellent CoV (< 2.5%). ICCs were good for all parameters (> 0.77), in particular for all PC-MRI and T2* measures (> 0.93).

**Table 3:** Baseline magnetic resonance imaging flow, perfusion, and transverse relaxation time (T2*) data for healthy participants and patients with compensated cirrhosis (CC)

Parameter		Healthy subjects (HS)		Compensated cirrhosis (CC)		Baseline HS versus CC *p* value	ICC
		Baseline	CoV (%)	Baseline	CoV (%)		
Portal vein	Area (mm^2^)	94.4 ± 25.5	7.3 ± 4.4	138.5 (92.4–141.7)	3.8 ± 2.6	0.063	0.984
	Velocity (cm/s)	13.2 ± 2.7	9.4 ± 7.6	13.7 ± 2.9	4.9 (4.2–5.6)	0.694	0.924
	Flux (mL/s)	12.0 ± 3.1	6.8 (5.7–11.0)	16.3 ± 4.5	5.7 ± 1.9	0.022^a^	0.979
Hepatic artery	Area (mm^2^)	24.6 ± 4.8	5.5 (3.9–7.9)	20.7 (19.8–23.4)	5.7 (2.7–8.5)	0.278	0.942
	Velocity (cm/s)	18.0 ± 3.5	9.4 (7.8–19.4)	16.2 (11.5–24.2)	7.9 ± 3.5	0.720	0.985
	Flux (mL/s)	4.5 ± 1.5	15.4 ± 8.5	3.5 (2.8–5.0)	7.5 ± 4.5	0.315	0.975
Total hepatic blood flow (mL/s)		16.2 ± 4.1	8.4 (5.6–9.5)	20.9 ± 5.0	5.5 ± 3.6	0.038^a^	0.972
Azygos vein	Area (mm^2^)	21.6 ± 6.3	6.0 (4.3–7.4)	20.8 (18.7–33.1)	2.5 (1.0–3.5)	0.635	0.985
	Velocity (cm/s)	10.1 ± 4.9	9.3 ± 4.4	13.4 ± 3.7	7.1 ± 5.0	0.144	0.977
	Flux (mL/s)	2.0 ± 0.6	14.7 ± 9.7	2.9 (2.3–5.0)	8.5 ± 5.2	0.042^a^	0.994
Liver perfusion (mL/100 g/min)		212.9 ± 53.9	11.2 ± 12.5	219.6 ± 75.5	12.5 ± 9.1	0.826	0.773
Arrival time of blood to liver (ms)		545 (499–604)	17.1 (4.9–41.3)	367 ± 89	26.1 ± 17.2	0.034^a^	0.793
T2* (ms)	Mode	17.8 ± 3.7	2.5 ± 1.3	14.5 ± 5.3	1.4 (1.3–2.7)	0.126	0.996
	FWHM	8.8 ± 2.3	4.4 (3.8–6.1)	7.8 ± 2.1	13.1 ± 10.1	0.294	0.930

Data are mean ± standard deviation for normally distributed data or median and interquartile range for non-normally distributed data

*CoV* coefficient of variation, *ICC* intra-class correlation (ICC)

^a^Statistical significance

### Postprandial response

**Fig. 3:**
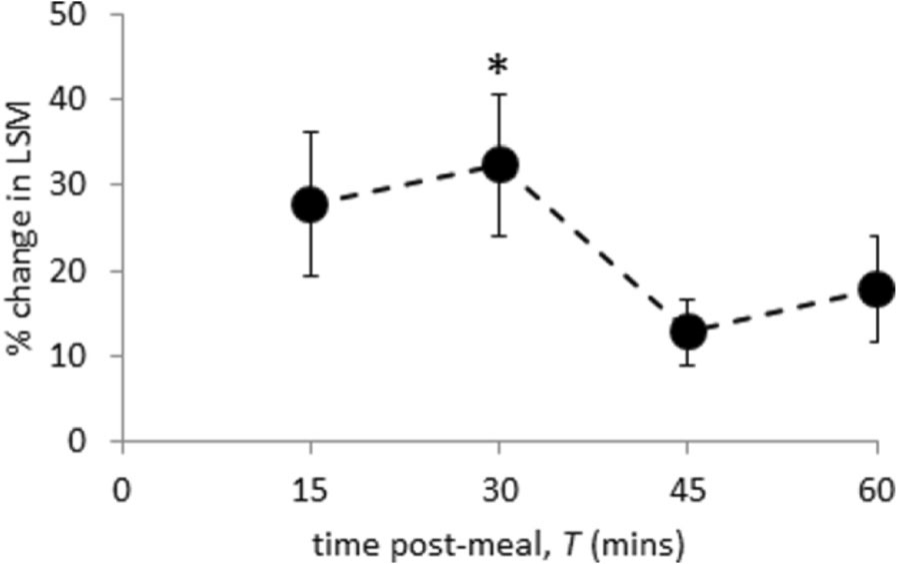
Percentage change in liver stiffness measurement (LSM) from baseline, following meal ingestion (mean ± standard error of the mean) in healthy participants. Comparison of each time point with baseline, *p < 0.05. *T* time

Figure 2 shows serum levels at baseline and *T* = 70 min. In healthy participants, there was no change in levels of ALT, ALP and GGT, whilst the reduction in serum bilirubin by 1.1 μmol/L was significant. In patients with CC, serum levels of ALT, ALP and GGT significantly decreased postprandial, with no change in serum bilirubin level. Among healthy participants there were no postprandial changes in TIMP1 or P3NP concentration; however, there was an increase in hyaluronic acid (HA) and therefore in ELF score. In patients with CC, levels of P3NP and TIMP1 decreased, but HA and the composite ELF score did not change. In healthy participants, the predicted postprandial increase in LSM was evident (Fig. 3), with the maximum increase from baseline at *T* = 30 min (*p* = 0.003).

**Fig. 4:**
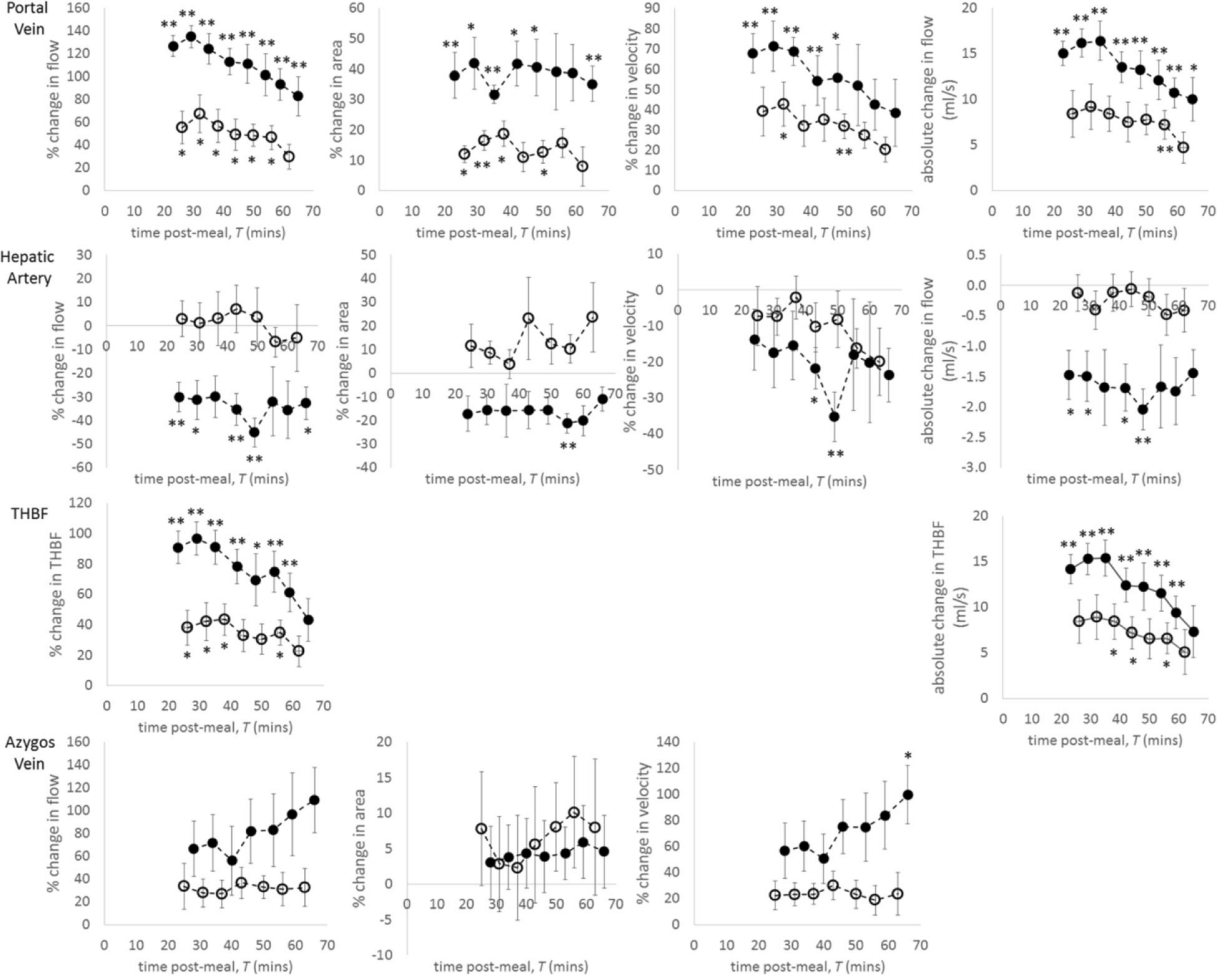
Percentage change in flow, area and velocity in the portal vein, hepatic artery, total hepatic blood flow (THBF) and azygos vein following meal ingestion in healthy participants and patients with compensated cirrhosis (mean ± standard error of the mean). Absolute change in flow is also given. Filled circles indicate healthy participants and open circles indicate patients with compensated cirrhosis. Comparison of each time point with baseline, *p < 0.05, **p < 0.01. *T* time

Figure 4 shows the change from baseline in postprandial blood flow measures. Maximum increase in portal vein flow from baseline occurred at about *T* = 30 min and was considerably larger in healthy participants (137 ± 26%, *p* < 0.001) than in patients with CC (67 ± 50%, *p* = 0.014). This increase resulted from the rise in portal vein velocity and vessel dilation (Fig. 4). In healthy participants, hepatic artery flow decreased postprandial (30 ± 18% reduction at *T* = 24 min, *p* = 0.008), which persisted during the study period. In contrast, there was no postprandial change in patients with CC. Postprandial vasoconstriction of the hepatic artery (16–21% during the postprandial study period) coupled with a reduction in blood flow velocity (35 ± 18% reduction at *T* = 49 min, *p* = 0.008) led to the reduced hepatic artery flow observed in the healthy participants. Postprandial THBF increased in both groups, with a larger increase in healthy participants (Fig. 4).

In healthy participants, azygos vein flow increased continually for the entire postprandial study period, reaching 109 ± 64% above baseline. This rise was driven by the continual increase in azygous blood flow velocity (0.032 at *T* = 65 min). There was no change in the area of the azygos vein in either group. In the patients with CC, azygos blood velocity, and therefore flow, increased at *T* = 25 min (34 ± 56% and 22 ± 31% for flow and velocity respectively) and remained elevated for the study duration, although this did not reach significance.

**Fig. 5:**
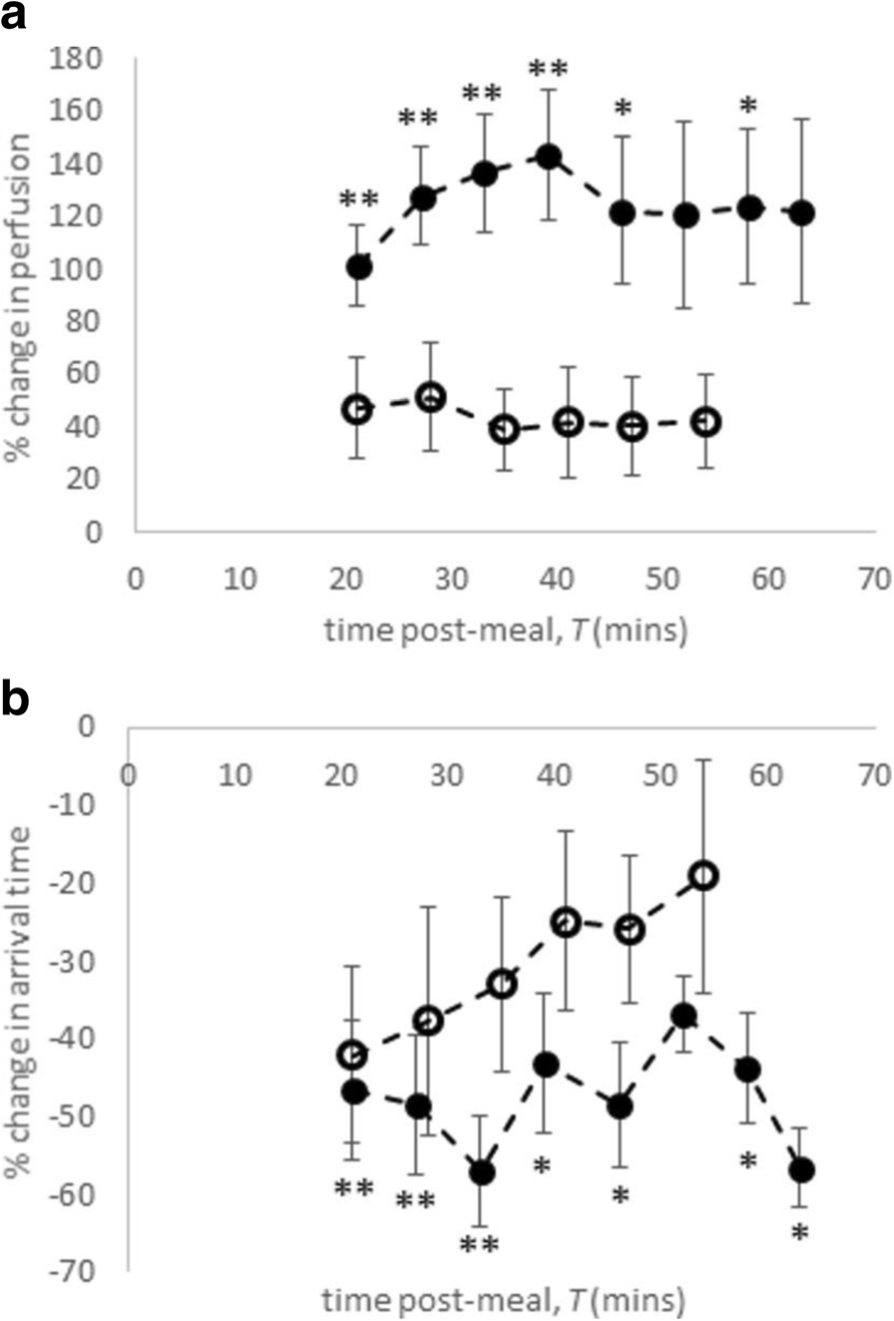
Percentage change in liver tissue perfusion (**a**) and blood arrival time (**b**) to the liver following meal ingestion in healthy participants and patients with compensated cirrhosis (mean ± standard error of the mean). Filled circles indicate healthy participants and open circles indicate patients with compensated cirrhosis. Comparison of each time point with baseline, *p < 0.05, **p < 0.01. *T* time

Liver tissue perfusion increased postprandial in both groups (Fig. 5), with a greater increase in healthy participants than in patients with CC. In both groups this increase was sustained during the postprandial study period. A maximum rise from baseline was seen in healthy participants at *T* = 39 min (138 ± 75%, *p* < 0.001) and in the patients with CC at *T* = 28 min (51 ± 58%). In both groups, increased perfusion was associated with a shorter arrival time of the labelled blood to the liver tissue. Reduced arrival time was sustained throughout the postprandial study period for the healthy participants (40–60% below baseline) whilst in patients with CC there was an initial decrease at *T* = 21 min (42 ± 32%) before returning towards baseline.

**Fig. 6:**
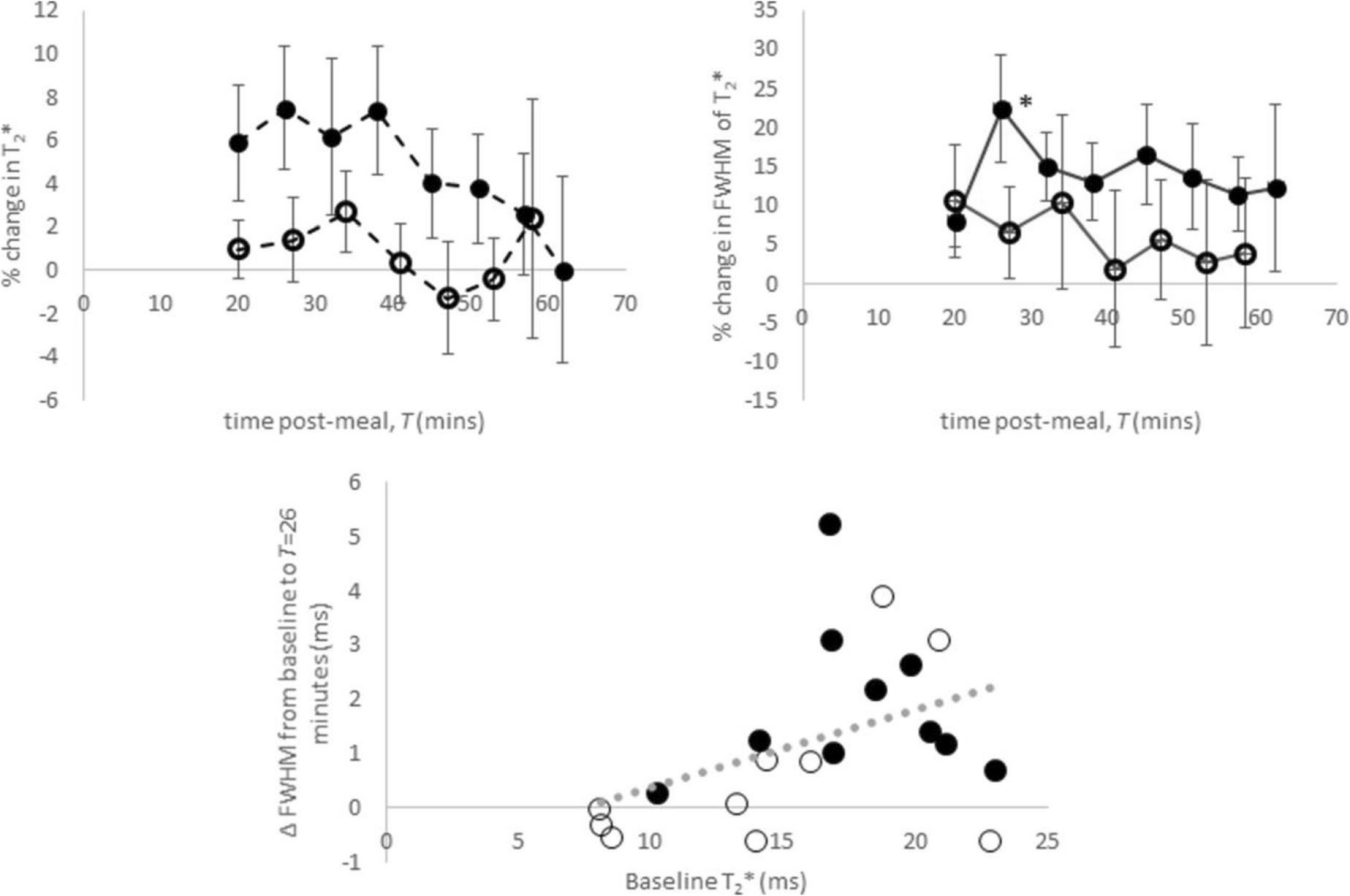
Percentage change in transverse relaxation time (T2*) following meal ingestion in healthy participants and patients with compensated cirrhosis (CC) (mean ± standard error of the mean). Filled circles indicate healthy participants and open circles indicate patients with compensated cirrhosis. Comparison of each time point with baseline, *p < 0.05

Both groups showed a trend for a postprandial increase in liver T2* (Fig. 6), with a larger maximum increase in healthy participants (7 ± 9% at *T* = 26 min), compared with patients with CC (3 ± 7% at *T* = 34 min). The FWHM of T2* increased significantly in healthy participants at *T* = 26 min (22 ± 21%), but no change was seen in patients with CC.

## Discussion

Noninvasive, quantitative MRI has been used to measure dynamic changes in hepatic and collateral flow, liver tissue perfusion and oxygenation in both healthy participants and patients with CC following a test meal. Baseline serum levels, ELF scores and LSM are in line with those reported in the literature for healthy participants and patients with CC [35, 36] Baseline MRI measures were highly reproducible in healthy participants and patients with CC, suggesting these noninvasive measures are feasible for assessing dynamic changes following a meal. We have recently demonstrated the validation of MRI measures in compensated cirrhosis against biopsy, indocyanine green clearance and clinical outcomes [37]. There has now been a number of comparative studies of reproducibility between PC-MRI and Doppler ultrasound, for example, demonstrating better PC-MRI 1-year reproducibility and reduced variability (combined total liver blood flow CoV of 18% for PC-MRI versus 33% for Doppler ultrasound) [12].

The reduction in serum bilirubin in healthy participants is unlikely to represent clinical significance as all values are well below the upper limit of the normal range (approximately 30 μmol/L). The postprandial rise in serum HA in healthy participants has been attributed to a manifestation of a metabolic challenge leading to a stress response in the liver [38]. The postprandial increase in LSM at *T* = 30 min in our healthy participants has previously been observed in patients with varying stages of liver fibrosis [17].

Our MRI measures showed postprandial portal vein flow increased in healthy participants, but was blunted in patients with CC during the entire study period, in agreement with Doppler ultrasound [39–41] and four-dimensional flow MRI [16] studies. The postprandial reduction in hepatic arterial flow in healthy participants, not seen in patients with CC, is also in keeping with Doppler ultrasound studies where a postprandial increase in hepatic artery resistance index diminished with the increasing fibrosis [24, 42]. The postprandial increase in THBF is dominated by the increase in portal vein flux, and the reciprocal changes in the portal vein and hepatic artery flow seen in our study are likely to reflect the hepatic artery buffer response, which regulates THBF. Multiple portal vein PC-MRI studies have demonstrated reduced portal vein flow in chronic liver disease and portal hypertension [10, 20]. Portal vein PC-MRI has also been used to demonstrate the expected postprandial increase in portal vein flow and following trans-jugular intrahepatic portosystemic shunt procedures [10].

A greater postprandial increase in azygos flow was seen in healthy participants compared with patients with CC. Here, the maximum rise occurred at the final postprandial time point (*T* = 65 min). Previously, postprandial azygos vein flow measured with PC-MRI in healthy participants was shown to increase by 38% at 30–40 min [20], but the continued increase in azygos vein flow at *T* = 65 min seen here in healthy participants has not been reported. Thus, we speculate this reflects the persistently elevated portal vein flow. However, in patients with CC, postprandial azygos blood flow was significantly increased after 20 min using four-dimensional flow MRI [16] and at 30 min but not at 45 min measured invasively using a thermal dilution catheter [2].

The postprandial rise in liver perfusion was blunted in patients with CC compared to healthy participants. This reflects the blunting of the postprandial portal venous flow in patients with CC, which is not compensated by the hepatic artery flow. Recently, arterial and portal venous perfusion of the liver was studied using MRI in healthy participants [21], with only a postprandial increase in venous perfusion observed.

In healthy participants, there was a trend for a postprandial increase in liver T2* and significant postprandial increase in the FWHM of T2*, indicating a capacity for change not seen in patients with CC. T2* of the liver is influenced by both iron [43] and oxygen content, here the dynamic increase in liver T2* reflects a change in the latter from either blood flow or oxygen consumption changes. Previously, an increase in T2* following glucose administration in healthy participants has been shown, although contributions from blood flow were not measured [44]. Hepatic oxygen uptake is reduced in cirrhosis [45] which may explain why there was no postprandial change in T2* in patients with CC.

Studying the dynamic changes after a meal could offer insight into key pathophysiological changes (hepatic artery buffer response and liver perfusion), which may have critical contributions to clinical management. Although the significance of hepatic artery buffer response in patients with CC is not completely understood, it is hypothesised that the increase in hepatic arterial flow in a low portal venous flow state sustains oxygen delivery to the liver and thus has a protective effect on organ function [46]. We propose that studying postprandial change in liver and collateral haemodynamics will aid individualised treatment strategies for portal hypertension and hence reduce the rate of treatment non-response. Haemodynamic change following a meal challenge has been utilised as a model to study the effects of novel treatment for portal hypertension [28, 47, 48]. In patients with extensive collateralisation and greater postprandial rise of the collateral circulation, the efficacy of eradicating the varices as the primary treatment strategy in reducing the risk of bleeding should be evaluated.

This study has some limitations. Disease severity was not homogeneous in this group of patients with CC and we do not have any hepatic venous-pressure gradient measurements. We did not specify the presence of diabetes mellitus in our inclusion/exclusion criteria. This may be of particular relevance in the two patients with non-alcoholic fatty liver disease, since significant changes in the postprandial ALT levels have been reported in participants with type 2 diabetes mellitus compared to healthy participants [38, 39]. The age range of the patients with CC was significantly higher than that of the healthy participants, and two participants were scanned in the afternoon, rather than in the morning as were the other participants. LSM were not possible in the patients with CC as sufficient valid measures could not be obtained before and after the meal challenge due to multiple technical factors relating to the Fibroscan equipment. It was not feasible to also dynamically measure gastric emptying or intestinal motility, which has been reported to differ between patients with CC and healthy participants [49–51].

In conclusion, noninvasive MRI was performed dynamically to capture haemodynamic and oxygenation changes in the postprandial period from *T* = 20 to 65 min. This provides a unique opportunity to concurrently study the effects of potential novel treatments for portal hypertension on liver and collateral blood flow and liver perfusion and oxygenation.

## Abbreviations

ALP: Alkaline phosphate
ALT: Alanine transaminase
ASL: Arterial spin labelling
CC: Compensated cirrhosis
CoV: Coefficient of variation
ELF: Enhanced liver fibrosis
FWHM: Full width at half maximum
GGT: Gamma-glutamyl transferase
HA: Hyaluronic acid
ICC: Intra-class correlation
LSM: Liver stiffness measurement
MRI: Magnetic resonance imaging
P3NP: Amino terminal peptide of procollagen IIIP3
PC: Phase contrast
SD: standard deviation
T2*: Transverse relation time
THBF: Total hepatic blood flow
TIMP1: Tissue inhibitor of matrix metalloproteinase 1
ΔM: Perfusion weighted difference
